# Reliability and validity of spinal coordination patterns during treadmill walking in persons with thoracic spine pain – a preliminary study

**DOI:** 10.1186/1471-2474-14-345

**Published:** 2013-12-09

**Authors:** Jean Wessel, Michael R Pierrynowski, Kelly Pennell, Linda J Woodhouse

**Affiliations:** 1 School of Rehabilitation Science, McMaster University, 1400 Main St. West, Hamilton, ON L8S 1C7, Canada; 2 Canadian Memorial Chiropractic College, 6100 Leslie Street, Toronto, ON M2H 3 J1, Canada; 3 Department of Physical Therapy, University of Alberta, Edmonton, AB T6G 2G4, Canada

**Keywords:** Thoracic spine pain, Gait, Spinal coordination, Reliability, Validity

## Abstract

**Background:**

Persons with low back pain fail to show the same transition as healthy individuals from in-phase to anti-phase rotation of the thorax and pelvis as walking speed increases. The purpose of this study was to determine if the relative phase of the thorax and pelvis during walking was a reliable (within day test-retest) and valid measure for persons with thoracic pain.

**Methods:**

The time series motion of the spine over C7, T8 and sacrum were measured at five treadmill walking speeds (0.67, 0.89, 1.12, 1.34, 1.56 m/s) in 19 persons with thoracic spine pain and 19 healthy control subjects. After a 20 minute rest, all tests were repeated. The average relative phases of the transverse plane rotation between C7-T8, C7-sacrum and T8-sacrum during a one-minute walk were calculated. The standard error of measurement (SEM) and the intra-class correlation coefficient (ICC) were used to estimate test-retest reliability. Three-way repeated measures analyses of variance were performed to determine the influence of group, walking speed and session on the relative phases.

**Results:**

The minimum transverse plane motion amplitudes, across all participants and speeds, for the C7-T8, C7-sacrum, and T8-sacrum were 2.9, 5.1 and 2.8 degrees, respectively. The C7-T8 relative phase changed little with speed. The C7-sacrum and T8-sacrum relative phases showed increases as subjects walked faster, but both groups had similar patterns of change. Only the C7-T8 relative phase at 0.67 and 0.89 m/s exhibited good reliability (ICC > 0.80, SEM 4.2-5.7, no significant time effects) for both groups. The C7-T8 and T8-sacrum relative phases demonstrated significant group by speed effects.

**Conclusions:**

The C7-T8 relative phase showed reasonable reliability and some discrimination between groups, but changes in response to walking speed were small. The T8-sacrum relative phase showed some discriminative ability, but reliability was not adequate.

## Background

Thoracic spine pain is commonly reported in the general population [1,2], and is more prevalent in females and adolescents [3,4]. A systematic review by Briggs and colleagues [4] estimated that the 12-month prevalence for thoracic spine pain in the general adult population is 15.0-34.8%, partially dependent on the operational definition of thoracic spine pain used in the various studies [4]. Although thoracic spine pain is common, it is not as prevalent as neck and low back pain in the adult population, possibly due to the inherent stability of the thoracic spine, enhanced by its articulations with the rib cage [5].

Presently there are no standardized measures for the outcome evaluation of persons with thoracic spine pain. Clinicians typically rely on static range of motion (ROM) of the spine and self-report measures of pain and function [6]. However, the value of measuring spinal ROM remains in question. Some studies show poor reliability [7], particularly with rotation [8]; little relationship to other measures of impairment, self-report function or response to treatment [9,10]; and inadequate ability to discriminate between normal and abnormal [11]. Lamoth and colleagues [12] found that the amplitude and spectral content of pelvic and thoracic rotations during gait were the same in persons with and without low back pain. However, the coordination of these rotations differed in the two groups. They concluded that coordination patterns might be useful in measuring the ‘quality of movement’ in persons with back pain. Self-report and performance measures are not always highly correlated and may respond quite differently to treatment. It would be useful to have a standardized, objective measure to evaluate change in persons with thoracic pain. Such a measure must be reliable and be different in healthy persons and those with thoracic pain.

**Figure 1:**
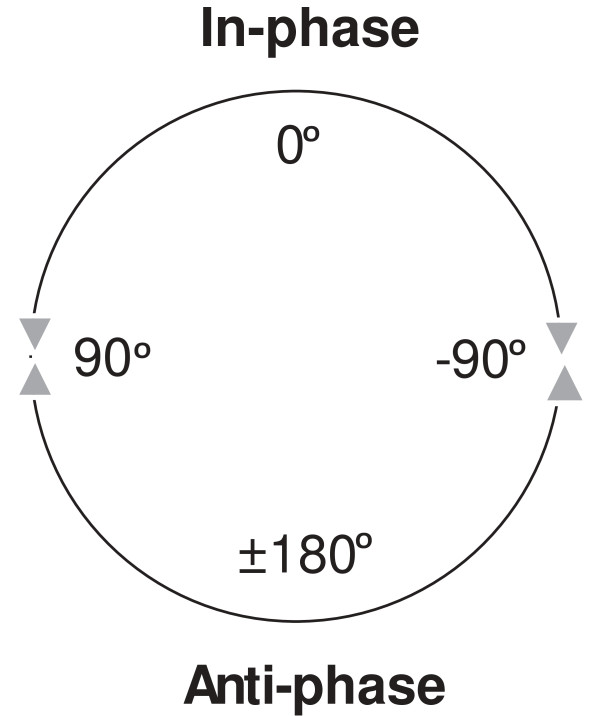
Illustration of relative phase – in-phase and anti-phase.

The assessment of spinal coordination patterns during walking may provide a means to objectively measure abnormalities associated with thoracic spine pain. One method to quantify spinal coordination is to examine the relative transverse plane rotation between two parts of the spine. Consider the transverse plane rotations of the thorax and pelvis. If *both* the thorax and pelvis are rotating clockwise (or counter-clockwise), they are in-phase. This can also be expressed as a relative transverse plane rotation between -90° and +90° (see Figure 1). If the thorax is rotating in the opposite direction to the pelvis, the rotations are anti-phase (bottom part of Figure 1). In healthy subjects, transverse plane rotations of the thorax and pelvis during walking at slow speeds are in-phase, but as walking speed increases, the transverse plane rotations shift towards anti-phase [13,14]. Persons with movement disorders such as stroke [15] or Parkinson’s disease [16], as well as persons with low back pain [12,17,18] failed to show the same shift to anti-phase coordination at higher walking speeds. Cox and colleagues [19] found that measures of spinal coordination were completely independent of self-assessment in persons with low back pain, and felt that they might provide information that would be complementary to the clinical examination. Perhaps measures of spinal coordination will also provide information that would help in the evaluation of treatment of persons with thoracic pain.

The purpose of this study was to estimate the reliability and determine the discriminative ability of the coordination (relative phase) of transverse plane rotation between C7 and T8, between C7 and sacrum and between T8 and sacrum during walking in persons with thoracic spine pain. If persons with thoracic pain were to respond in the same manner as persons with low back pain, we expected that the coordination of T8-sacrum would move from in-phase to anti-phase as walking speed increased (i.e. relative phase would increase), but the changes would be smaller in persons with thoracic pain compared to healthy subjects. We were unsure how C7-T8 and C7-sacrum would respond to increased speed. However, if the pain in the thoracic spine were responsible for changes in coordination, we expected there to be differences in the coordination patterns of C7-T8 and C7-sacrum in the control and case subjects.

## Methods

### Study participants

Nineteen adults with non-specific thoracic spine pain were included as case subjects in this study. Thoracic pain was defined as pain across the posterior aspect of the trunk in the area between T1 and T12 and persisting for greater than 3 months. Other eligibility criteria included: age 18 to 65 years; ambulation without a walking aid; willingness to attend the laboratory for approximately 2 hours of testing; and ability to read/write English. Exclusion criteria were: thoracic pain of traumatic, visceral, or structural origin (e.g. moderate to severe scoliosis, hyper-kyphosis, as determined by observation); thoracic pain with neurological symptoms; spinal tumours or infections; previous back surgery; neurological and/or musculoskeletal disorders unrelated to thoracic spine pain; low back pain and cardiovascular conditions. Although not all subjects had undergone medical examination, a chiropractor examined all volunteers for obvious structural changes and neurological symptoms. Other information on exclusion criteria was obtained by self-report. Volunteers with a BMI greater than 35 kg/m^2^ were excluded as it was expected that increased BMI could alter gait and influence spinal motion. Individuals who reported neck pain were not excluded as long as their predominant pain was reported to be in the thoracic spine.

The healthy control subjects were 19 adults without thoracic spine pain, matched with case subjects for age (within five years) and gender. All other eligibility criteria for the control subjects were the same as those for the cases.

Case subjects were recruited from local physiotherapy, chiropractic, athletic therapy, and sports medicine clinics and from the university community where the research took place. Control subjects were recruited from university staff and students, and from the surrounding communities. Methods of recruitment included email, posters, newspaper advertisements, and direct referrals from various clinics. Recruitment was performed without regard to racial group, occupation, or socio-economic status. All subjects provided written informed consent and the study received approval from the Hamilton Health Sciences/McMaster Health Sciences Research Ethics Board.

### Study design and protocol

This study used a 2-group, repeated measures mixed-effect model to compare case subjects with control subjects, and to determine the reliability of the spinal coordination measures. All subjects attended the laboratory on one occasion only, for approximately 2 hours. They were tested twice for all measures with a 20 minute break between test sessions to avoid fatigue. All data collection was performed by the same investigator (KP).

At the beginning and end of each test session, subjects rated their present pain in the thoracic spine. Subject’s age gender, height, weight, current medications, presence or absence of neck pain, and duration of thoracic spine pain were collected. Height and weight measurement were used to calculate the participant’s BMI. Subjects walked at their “normal’ pace (neither fast nor slow) for a known distance in the corridor so that their comfortable walking speed (CWS) could be calculated. The CWS was used to compare the walking ability of the two groups. The subjects practiced walking on the motorized treadmill at 1.12 m/s for one minute prior to the commencement of data collection for the calculation of relative phase.

### Measures

The measures for this study were the continuous relative phases of the transverse plane rotations of three divisions of the spine (C7-T8, C7-sacrum and T8-sacrum). Subjects walked on a treadmill (True S.O.F.T System 500, True Fitness Technology, O’Fallon, Missouri) at varying walking speeds (0.67, 0.89, 1.12, 1.34, and 1.56 m/s) that were presented in random order. At each speed, subjects had one minute of practice and then data were collected for one minute. All subjects were instructed to walk as naturally as possible in the middle of the treadmill belt without using the handrails.

**Figure 2:**
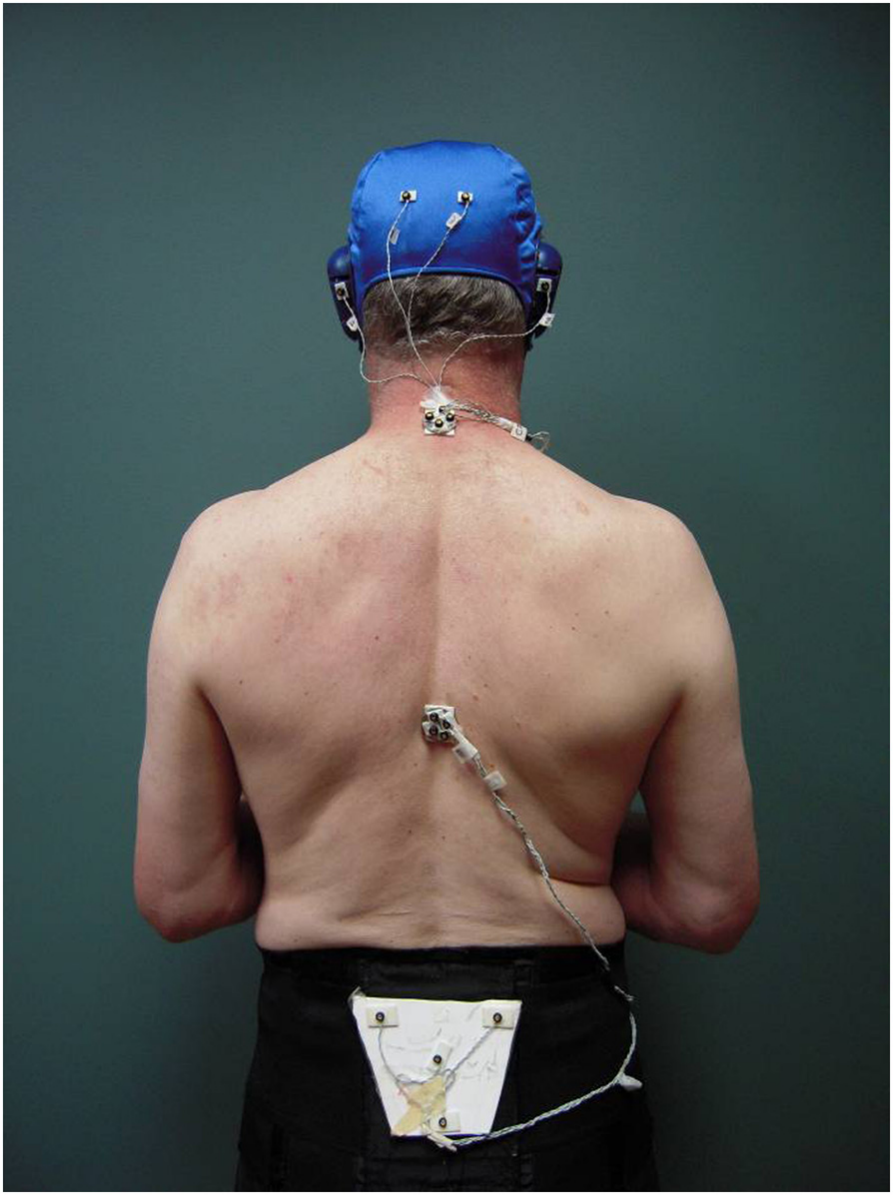
**Subject with markers attached to head, and over C7, T8 and sacrum.** Note: data from head not used in present study.

During the walks a kinematic data acquisition system (OptoTrak™, Northern Digital Inc., Waterloo, Canada at 100 Hz; or UltraTrak Pro™, Polhemus, Colchester, Vermont at 60 Hz) positioned behind or to the side of the subject recorded the displacement of a four diode, 28 mm rigid disk (OptoTrak™) or the location and orientation of one 26 mm rigid sensor (UltraTrak Pro™) securely attached, using double-sided tape, to the skin over C7 and T8 (see Figure 2). The 3D motions of the 4 diode markers on each disk were processed to provide location and orientation of the disk’s center [20] providing outputs comparable to those available from the sensors. Skin-attached markers have been successfully used to record the motion of the spine during gait [21]. The sacrum disk/sensor was embedded within an elastic belt worn around the subject’s upper pelvis to provide an estimate of pelvic motion.

The abilities of the OptoTrak™ and Polhemus systems to measure relative angular position were 0.67° and 1.2° respectively [22,23]. Since the expected transverse plane rotation of the spine was 10° [24], each system provided data with an adequate signal-to-noise ratio. A diode cluster or a sensor was also attached to the head but was not used for this study.

The third derivative of the transverse plane rotation of the pelvis disk/sensor measured during each trial was used to estimate the start of each gait cycle within that trial. Specifically, the even (or odd) set of maxima in the third derivative of the time function of the transverse plane rotation provides good estimates of the heel-ground contacts [25].

Within each disk/sensor an anterior-posterior (AP) vector was defined when the subject stood in a reference (anatomical position) posture. During treadmill walking the transverse plane angles of the three AP vectors relative to their reference posture were calculated using vector algebra informed by geometry. Within each gait cycle, within each trial, the transverse plane angles were least-squares fitted to a sine wave with parameters amplitude and phase. The phase specifies where in the gait cycle (-180, 180°) the sinusoidal wave crosses from negative to positive. Subtraction of one phase angle from another yields the relative phase between transverse plane rotations. A positive relative phase indicated that the superior disk/sensor rotated counter-clockwise relative to the inferior disk/sensor when viewed from above.

There were 15 relative phase measures (C7-T8, C7-sacrum, T8-sacrum at 5 walking speeds) obtained for each subject. The criterion measure for each was the relative phase averaged across all the gait cycles that were recorded during the one minute of data collection.

A 10 cm visual analogue scale (VAS) with anchors of ‘no pain’ and ‘worst possible pain’ was used by the subjects to rate the intensity of their thoracic pain [26].

### Statistical analysis

All statistical procedures were performed using SPSS Version 16.0 (SPSS Inc.; Chicago, Illinois), and included descriptive statistics and tests for normality of the data. T-tests were used to compare the characteristics of the case and control subjects. The pain VAS of the case subjects was examined by means of a two-way ANOVA with repeated measures on both session and time within session.

The standard error of measurement (SEM) and the type (2, 1) intraclass correlation coefficient (ICC) [27] were used to estimate test-retest reliability of the relative phase for C7-T8, C7-sacrum and T8-sacrum at the five walking velocities. To account for sampling variation, a 95% confidence interval (95% CI) was constructed about the ICC point estimates.

Three-way ANOVAs with repeated measures on two factors were performed to determine the influence of group, session, and walking speed for the relative phases of C7-T8, C7-sacrum and T8-sacrum. Differences were deemed to be statistically significant at *P* ≤ 0.05. Because this was a preliminary study, no correction factor was used to account for multiple comparisons. Following primary analyses, post hoc contrasts were performed to further assess differences between speeds. If the measure of relative phase were able to discriminate between persons with and without thoracic pain, a significant group by speed interaction was expected. Significant time effects would suggest poor reliability.

## Results

### Group characteristics

Forty individuals volunteered to participate in this study. Data collection was completed for 38 subjects; 19 volunteers with thoracic spine pain and 19 healthy gender-matched volunteers. The majority of subjects were matched for age (within 5 years) with the exception of two subjects due to limitations associated with recruitment and time constraints. One volunteer was not accepted for the study because the BMI was greater than 35 kg/m^2^. The data from one subject could not be used due to equipment failure during testing. Characteristics of the study participants are presented in Table 1. T-tests showed no significant differences in the characteristics of the two groups (*P*: 0.12 to 0.96). There were no changes in the pain VAS of the case subjects from pre to post testing in either session (Session 1: 2.4 ±1.8 to 2.2±2.0 cm; Session 2: 1.8±1.8 to 2.1±1.8 cm). However, the mean pain was higher at Session 1 compared to Session 2 (session: df 1, F 9.9, *P* <0.01, pre/post: df 1, F 2.2 *P* 0.13, interaction: df 1, F 2.4, *P* 0.11). No control subjects reported experiencing pain at any time.

**Table 1:** Characteristics of case and control subjects

**Characteristic**	**Case subjects mean (SD)**	**Control subjects mean (SD)**
**Age (years)**	32.0 (11.8)	30.6 (9.0)
**Height (cm)**	168.9 (8.3)	169.0 (6.9)
**Mass (kg)**	62.9 (10.1)	63.0 (9.2)
**BMI (kg/m**^**2**^**)**	22.0 (2.5)	22.1 (2.9)
**CWS (m/s)**	1.45 (0.04)	1.38 (0.02)
**Duration of TSP (months)**	43.1 (35.3)	
	**n (%)**	**n (%)**
**Gender Female**	17 (90%)	17 (90%)
**Male**	2 (10%)	2 (10%)
**Presence of neck pain**	8 (42%)	

CWS = comfortable walking speed.

TSP = thoracic spine pain.

### Relative phase reliability and validity

The minimum transverse plane motion amplitudes, across all participants and speeds, for the C7-T8, C7-sacrum, and T8-sacrum, were 2.9, 5.1 and 2.8 degrees, respectively. These amplitudes were of sufficient size to fit a sine wave to the data to estimate the relative phases.

**Figure 3:**
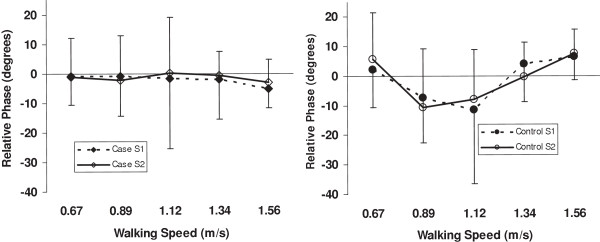
**C7-T8 relative phase for case and control subjects at 5 walking speeds.** S = session.

**Figure 4:**
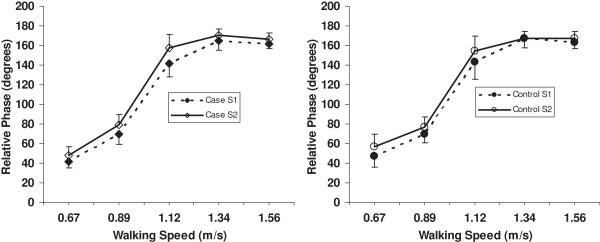
**C7-sacrum relative phase for case and control subjects at 5 walking speeds.** S = session.

**Figure 5:**
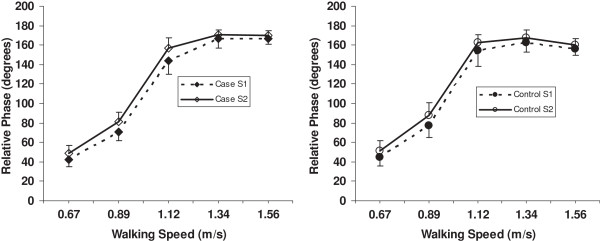
**T8-sacrum relative phase for case and control subjects at 5 walking speeds.** S = session.

All the relative phase data were normally distributed as determined by the Kolmogrov-Smirnov test except for T8-sacrum relative phase at 0.67 and 1.56 m/s for the case subjects at Session 1. Therefore, we continued with the parametric statistical tests as planned, and performed additional non-parametric tests on T8-sacrum relative phase at these two speeds. The relative phases for C7-T8, C7-sacrum and T8-sacrum are depicted in Figures 3, 4 and 5, respectively. The C7-T8 relative phase changed little with increasing walking speeds, that is, the coordination of the transverse plane rotation of C7 and T8 remained relatively in-phase. On the other hand, the C7-sacrum and T8-sacrum relative phases increased markedly from 0.67 to 1.12 m/s and then tended to plateau. The C7-sacrum and T8-sacrum relative phases were close to complete anti-phase (180 degrees) at the three fastest speeds. Standard deviations were higher than the mean values at all speeds for C7-T8 relative motion.

The results of the reliability analyses are presented in Table 2. The SEMs of relative phase ranged from 2.0° (T8-sacrum, control group at 1.56 m/s) to 11.8° (C7-T8, control group at 1.12 m/s). Although some of the point estimates of the ICCs were above the 0.75 considered necessary for good reliability [28], only the C7-T8 showed narrow CIs.

**Table 2:** Standard error of measurement (SEM) and intraclass correlation coefficients (ICC) of relative phase of C7-T8, C7-sacrum and T8-sacrum

		**Case subjects**			**Control subjects**		
**Speed (m/s)**		**C7-T8**	**C7-sacrum**	**T8-sacrum**	**C7-T8**	**C7-sacrum**	**T8-sacrum**
**0.67**	**SEM degrees**	4.2 (3.2–6.2)	2.5 (1.9–3.7)	2.6 (2.0–3.8)	4.5 (3.4–6.7)	2.8 (2.1–4.1)	2.8 (2.2–4.2)
	**ICC (CI)**	**.87 (.70–.95)**	.66 (-.08–.91)	.64 (-.09–.90)	**.88 (.68–.96)**	.70 (-.06–.93)	.74 (-.07–.93)
**0.89**	**SEM degrees**	5.3 (4.0–7.9)	3.5 (2.6–5.2)	4.33.3–6.4)	5.7 (4.3–8.4)	4.0 (3.0–5.9)	3.0 (2.3–4.4)
	**ICC (CI)**	**.86 (.68–.95)**	.66 (-.08–.91)	.49 (-.10–.82)	**.89 (.72–.96)**	.68 (-.04–.90)	.71 (-.06–.93)
**1.12**	**SEM degrees**	6.3 (4.8–9.4)	4.4 (3.3–6.5)	6.8 (5.1–10.0)	11.8 (8.9–17.4)	4.7 (3.5–6.9)	9.4 (7.1–13.9)
	**ICC (CI)**	**.91 (.79–.97)**	.55 (-.06–.87)	.44 (-.11–.78)	.70 (.38–.87)	.74 (-.07–.93)	.41 (-.01–.71)
**1.34**	**SEM degrees**	8.8 (6.7–13.0)	3.9 (3.0–5.8)	7.1 (5.4–10.6)	6.9 (5.2–10.2)	5.2 (3.9–7.7)	4.9 (3.7–7.2)
	**ICC (CI)**	.36 (-.11–.70)	.64 (.02–.87)	.08 (-.32–.48)	.66 (.30–.85)	.64 (.27–.85)	.65 (.21–.86)
**1.56**	**SEM degrees**	5.1 (3.9–7.6)	2.0 (1.5–2.9)	4.1 (3.1–6.1)	4.8 (3.6–7.1)	3.7 (2.8–5.4)	2.0 (1.5–3.0)
	**ICC (CI)**	.47 (.06–.76)	.65 (-.09–.90)	.49 (.08–.76)	.66 (.30–.85)	.55 (.02–.82)	.78 (.01–.94)

SEM = standard error of measurement.

ICC = intraclass correlation coefficient.

CI = 95% confidence interval; CIs for SEM and ICC in parentheses.

ICCs above 0.85 with narrow CIs are indicated in bold.

The ANOVA (Table 3) revealed significant group by speed effects for the C7-T8 and T8-sacrum relative phases, but not for C7-sacrum relative phase. A time main effect and interactions involving time were also significant for C7-sacrum and T8-sacrum. Post hoc analysis of the group by speed effect for C7-T8 indicated that the control group had an increase (in the negative direction) in relative phase between 0.67 and 0.89 m/s, and a decrease after 1.12 m/s, while the case group had no change in relative phase across speeds. For the T8-sacrum relative phase, the significant group by speed interaction was due to the case group having a greater increase from 1.12 to 1.34 m/s and less of a decrease from 1.34 to 1.56 m/s compared to the control group. The post hoc contrasts of the group by speed by time for C7-sacrum were significant for the two lower speeds and the two higher speeds. As can be seen from Figure 4, the slope of the change between 0.67 and 0.89 m/s was greater at Session 2 compared to Session 1 for the case subjects, but just the opposite for the control subjects. Between 1.34 and 1.56 m/s the change for the case group was similar at both sessions, whereas the control group had a decrease in relative phase at Session 1 and a slight increase at Session 2.

**Table 3:** Summary of analyses of variance for relative phase of C7-T8, C7-sacrum and T8-sacrum

**Source**		**C7-T8**		**C7-sacrum**		**T8-sacrum**	
	**df**	**F**	***P***	**F**	***P***	**F**	***P***
**Group**	1, 36	0.1	.768	0.6	.441	0.3	.610
**Speed**	4, 144	2.4	.057	1226.6	<.001	1475.3	<.001
**Time**	1, 36	0.5	.491	419.4	*<.001*	156.6	*<.001*
**Group × Speed**	4, 144	3.5	**.009**	1.3	.268	6.8	**<.001**
**Group × Time**	1, 36	0.5	.493	5.1	*.030*	0.5	.496
**Speed × Time**	4, 144	2.0	.095	21.5	*<.001*	8.3	*<.001*
**Group × Speed × Time**	4, 144	1.4	.238	4.0	***.004***	1.2	.313

Significant effects involving group × speed interaction (*P* ≤ .05) are in **bold**.

Significant time effects (*P* ≤ .05) are in *italic*.

The results of the non-parametric tests for T8-sacrum are listed in Table 4. They confirm the differences between the speeds and the sessions. There were significant differences between groups at the higher speed only.

**Table 4:** Non-parametric tests for relative phase T8-sacrum at 0.67 and 1.56 m/s

**Comparison**	**Case subjects**				**Control subjects**		
		**X**^**2**^	**Kendall’s W**	***P***	**X**^**2**^	**Kendall’s W**	***P***
**Walking speeds**	T1	69.9	0.920	<0.001	62.8	0.827	<0.001
	T2	66.2	0.871	<0.001	62.0	0.816	<0.001
			**Wilcoxon Z**			**Wilcoxon Z**	
**T1 and T2**	0.67 m/s		-3.823	<0.001		-3.823	<0.001
	1.56 m/s		-2.052	0.04		-3.823	<0.001
	**Time 1**				**Time 2**		
**Groups (Mann Whitney U Test)**	0.67 m/s			0.452			0.418
	1.56 m/s			<0.001			<0.001

## Discussion

We believe this is the first study to investigate coordination patterns of the spine during walking in persons with thoracic spine pain. Generally the reliability of the relative phase was not good, as determined by the ICC, SEM and significant time effects. Only C7-T8 demonstrated reasonable reliability at the two slower speeds for both case and control subjects. The C7-T8 relative phase also showed some discrimination between groups, but the shift in relative phase with changes in speed was very small. The C7-sacrum and T8-sacrum relative phases showed the expected increases as subjects walked faster, but both groups had similar patterns of change.

Overall, the results indicate that the C7-T8 relative phase has the greatest promise as an outcome measure for persons with thoracic spine pain. This measure not only had good reliability at the lower speeds, but the group × speed interactions suggest that this relative phase has potential to discriminate between persons with and without thoracic spine pain. Although the relative phases were small at all velocities, the pattern of change between velocities was different for the case and control subjects, and different from the C7-sacrum and T8-sacrum relative phases seen in the present study and reported in the literature [13,29]. Investigators have noted that the walking speed of healthy subjects has little effect on the range of thoracic rotation [30] or its timing within the gait cycle [29,31]. The thoracic spine becomes stiffer [30], however, and this ‘normal’ response might be adequate to protect the thoracic spine of persons with pain in this area. In the present study, the case subjects may have had a stiffer thoracic spine even at the lower walking speeds. Increased activity of the spinal muscles contributes to thoracic stiffness [30], and could be the result of ‘guarding’ in those with thoracic pain.

It should be noted, that although there were significant group × speed effects for C7-T8 relative phase, the standard deviations for the measures were large compared to the mean values (see Figure 3), and sometimes greater than the mean difference between groups. Also, because the differences between groups were small, these differences were not much higher than the upper bounds of the 95% CI for the SEMs. Therefore, it would be important to try to decrease the measurement error even further if C7-T8 relative phase were to be considered as an outcome for studies of thoracic spine pain.

Bruijn and colleagues [29] reported that a change in the timing of pelvic rotation during the gait cycle (and not in thoracic rotation) was the major contributor to the increase in T6-pelvis relative phase with increases in walking speed in healthy subjects. Their results could help explain why relative phase changes with walking speed were different from control subjects in persons with low back pain (perhaps affecting pelvic rotation) but not in persons with thoracic pain (further away from the pelvis).

The change in C7-sacrum and T8-sacrum relative phases from Session 1 to 2 may be due to subjects’ increased comfort with treadmill walking by Session 2. It is known that stride length increases as individuals accommodate to the treadmill [32]. In the present study, subjects were given time at the beginning of the study and prior to the tests at each walking speed to practice walking on the treadmill. However, it is still possible that they were more at ease with the procedure during the second session and walked with greater stride length. Adding more time for familiarization with the treadmill might have improved reliability, but it would also increase the subjects’ total walking time and could lead to fatigue especially for the case subjects. It is unlikely that the equipment or procedure would contribute to systematic error because the two test sessions were separated by a short 20 minute break. Future studies could examine the effects of additional trials and sessions and longer accommodation periods on reliability.

Although the mean values for relative phase increased from Session 1 to 2, the patterns of coordination with respect to walking speed remained relatively constant. Comparing curve shapes of the two groups might provide better discrimination. This could be done using principal differential analysis [33].

The case group had relatively low pain scores at rest, and may have responded differently than a group with greater thoracic pain. In the present study, pain duration (≥ 3 months) was an inclusion criterion for case subjects but minimum pain intensity was not. Other measures of severity or impact, such as a disability or pain with activity, were also not included in this study. Future research could comprise more severely affected subjects or the examination of relative phase in subjects with varying degrees of pain and disability.

The protocol in the present study was slightly different from previous studies [13,17,34] that have demonstrated the discriminative ability of spinal coordination patterns. Lamoth and colleagues [17] presented six walking speeds sequentially to their subjects with no stopping between velocities. The speeds were presented in random order in the present study, and a habituation period of 1-minute was included at each speed. The increments between speeds were smaller in some previous studies [35]. It is possible that the shape of the relative phase changes would be more discriminating at the lower speeds if the increments were smaller. The relative phase tended to plateau at the higher speeds, suggesting that the highest walking speed was not required. The use of adaptive trials [36] may be useful to select walking speeds in regions of greatest change.

Limitations to this study included sample size, surface markers and the use of two different data collection systems. The sample size was calculated to identify a reliability of 0.9 with a lower confidence limit of 0.8. Based on unpublished data from our laboratory, we calculated that 27 subjects per group were required to show differences with a power of 0.82, and alpha of 0.05 and a beta of 0.20. Therefore, the study was underpowered to discriminate between groups. With surface markers, skin movement can cause some error in measurement. However, Bruijn and colleagues [29] determined that this effect was small when measuring the timing differences in the rotation of different parts of the spine. Although two different data collection systems were used in this study, they both have good accuracy as previously described [22,23].

Our study also did not completely isolate the thoracic spine. The markers at C7 and T8 captured the top two-thirds of the thoracic spine, but the lower marker included movement of the lumbar spine along with movement of the lower thoracic. It is possible that results between groups might have been different with the measurement of T12 or L1. However, Willems et al. [37] showed in a younger cohort (18–24 years) that the lower third of the thoracic spine has less transverse rotation movement than the T1-4 or T4-8 segments. Moreover, the lumbar spine and pelvis tend to move together (in-phase) at all walking speeds [17,18]. Nevertheless, we did not specifically capture the relative phase of the lower thoracic spine with the middle or upper components.

## Conclusions

This is the first study to evaluate the reliability and discriminative abilities of the relative phase spinal motion in persons with thoracic spine pain. The C7-T8 relative phase had good test-retest reliability at lower speeds for both case and control subjects. Group by speed interactions were observed for two of the relative phase measures (C7-T8 and T8-sacrum), suggesting these measures could potentially discriminate between persons with and without thoracic spine pain. The results from this study indicate that the C7-T8 relative phase has the greatest potential of the three relative phase measures for the assessment of individuals with thoracic spine pain.

## Competing interests

The authors declare that they have no competing interests.

## Authors’ contributions

All authors were fully involved in the design of the study, the interpretation of the data and the preparation of the manuscript. Data were collected by KP and processed by MP. Statistical analyses were performed by KP and JW. All authors read and approved the final manuscript.

## Pre-publication history

The pre-publication history for this paper can be accessed here:

http://www.biomedcentral.com/1471-2474/14/345/prepub
